# Superconductivity in (Ba,K)SbO_3_

**DOI:** 10.1038/s41563-022-01203-7

**Published:** 2022-02-28

**Authors:** Minu Kim, Graham M. McNally, Hun-Ho Kim, Mohamed Oudah, Alexandra S. Gibbs, Pascal Manuel, Robert J. Green, Ronny Sutarto, Tomohiro Takayama, Alexander Yaresko, Ulrich Wedig, Masahiko Isobe, Reinhard K. Kremer, D. A. Bonn, Bernhard Keimer, Hidenori Takagi

**Affiliations:** 1 Max Planck Institute for Solid State Research, Stuttgart, Germany; 2 Stewart Blusson Quantum Matter Institute, University of British Columbia, Vancouver, British Columbia Canada; 3 ISIS Facility, STFC Rutherford Appleton Laboratory, Oxon, United Kingdom; 4 Department of Physics & Engineering Physics, University of Saskatchewan, Saskatoon, Canada; 5 Canadian Light Source, University of Saskatchewan, Saskatoon, Canada; 6 Department of Physics, University of Tokyo, Tokyo, Japan

**Keywords:** Superconducting properties and materials, Electronic properties and materials

## Abstract

(Ba,K)BiO_3_ constitute an interesting class of superconductors, where the remarkably high superconducting transition temperature *T*_c_ of 30 K arises in proximity to charge density wave order. However, the precise mechanism behind these phases remains unclear. Here, enabled by high-pressure synthesis, we report superconductivity in (Ba,K)SbO_3_ with a positive oxygen–metal charge transfer energy in contrast to (Ba,K)BiO_3_. The parent compound BaSbO_3−*δ*_ shows a larger charge density wave gap compared to BaBiO_3_. As the charge density wave order is suppressed via potassium substitution up to 65%, superconductivity emerges, rising up to *T*_c_ = 15 K. This value is lower than the maximum *T*_c_ of (Ba,K)BiO_3_, but higher by more than a factor of two at comparable potassium concentrations. The discovery of an enhanced charge density wave gap and superconductivity in (Ba,K)SbO_3_ indicates that strong oxygen–metal covalency may be more essential than the sign of the charge transfer energy in the main-group perovskite superconductors.

## Abstract (web summary)

High-pressure synthesis is used to stabilize superconducting (Ba,K)SbO_3_, whose properties provide a fresh perspective on the origin of superconductivity in these types of materials.

## Main

Superconducting bismuthates, BaPb_1–*x*_Bi_*x*_O_3_ (BPBO)^1^ and Ba_1–*x*_K_*x*_BiO_3_ (BKBO)^2,3^, have attracted considerable research interest since their discovery more than three decades ago. The parent compound BaBiO_3_ (BBO) is known to be a non-magnetic, commensurate charge density wave (CDW) insulator. The CDW order is accompanied by a breathing octahedral distortion; that is, two octahedra with different sizes order in a three-dimensional chequerboard pattern^4,5^. As the CDW order is suppressed via chemical substitution of Bi with Pb or Ba with K (refs. ^6,7^), the compounds become superconducting up to a maximum *T*_c_ of 12 K in BPBO, and 30 K in BKBO. Numerous experiments have established that the mechanism of superconductivity is largely conventional; the pairing symmetry is *s*-wave^8^, and the oxygen isotope effect is consistent with the Bardeen–Cooper–Schrieffer (BCS) theory^9^, meaning electron–phonon interaction plays the important role in superconductivity. Nevertheless, the unexpectedly high *T*_c_ of BKBO, despite a rather low carrier density, has triggered various critical questions as to the driving mechanism and the correct model of the CDW order and superconductivity in these materials. For example, recent studies suggest that the additional consideration of long-range exchange interactions and many-body effects can be crucial for the quantitative description of the CDW gap^10,11^ as well as for superconductivity^12–14^.

Superconducting bismuthates have long been considered as archetypal candidates for unconventional superconductors in which an effective electron–electron interaction *U* is negative ^15,16^, leading to electron pairing in real as well as in momentum spaces^17,18^. The real-space pairing occurs in the parent compound BBO, which is argued to be a typical valence-skipping compound with unstable tetravalent bismuth (6*s*^1^) disproportionated into tri- (6*s*^2^) and pentavalent (6*s*^0^). As the charge disproportionation on the bismuth sites is suppressed via chemical doping and the bismuth valence starts to dynamically fluctuate, negative *U*, which causes pairing of two electrons in the Bi^3+^–O_6_ octahedra, could also pair them in *k*-space. The negative *U* model may provide a possible framework to understand superconductivity in the bismuthates as well as some chalcogenides^19^ with valence-skipping elements.

While the negative *U* model emphasizes the role of bismuth, an alternative model asserts the role of oxygen and its hybridization with bismuth^6,20–22^. This model’s foundation is that the charge transfer energy *Δ*_CT_ of the bismuthates is negative, as the on-site energy of the Bi 6*s* orbital is lower than that of the oxygen 2*p*, owing to the large scalar relativistic effect of heavy bismuth^23^. Consequently, electronic states around the Fermi level (which originate from the strongly hybridized spσ* states) show predominantly oxygen 2*p* character. This crucially modifies the preceding understanding of the CDW order in BBO; it should be described not by the charge disproportionation (6*s*^2^ + 6*s*^0^) but rather by the bond-length disproportionation as 6*s*^2^ + 6*s*^2^*L*^2^, where *L* denotes a ligand hole. Spectroscopic evidence supports the oxygen-hole model^24–26^. As the CDW order is suppressed, oxygen holes become delocalized, giving rise to superconductivity, possibly via strong electron–phonon coupling^21,27^. The importance of oxygen holes has previously been demonstrated in the Zhang–Rice model^28^ for cuprates, in which holes on the copper and oxygen sites form a strongly hybridized singlet state, highlighting their potential role in understanding the CDW order and high-*T*_c_ superconductivity in the bismuthates as well.

In spite of their scientific importance, a contrastive analysis of the effects of bismuth and oxygen has so far been limited due to lack of compounds analogous to the bismuthates. Perovskite antimonates are ideal candidates to study; antimony is isovalent to bismuth. Higher on-site energy of the Sb 5*s* orbital compared to the Bi 6*s* may enable us to tune the *Δ*_CT_ of the material from negative to almost zero or even positive; therefore the states around the Fermi level are expected to show stronger metal *s* character. First-principles calculations of a hypothetical primitive cubic perovskite BaSbO_3_ (BSO) have been previously conducted and compared with those of BBO^29–31^. The results clearly indicate that the Sb 5*s* level is well above the Bi 6*s* level, increasing *Δ*_CT_ from established negative values in BBO to higher energy values in BSO. It would therefore be enlightening to see the impact of this drastic change of *Δ*_CT_ on both superconductivity and CDW in antimonates, which should give us a hint to identify the key ingredient of high-*T*_c_ superconductivity in BKBO.

Several attempts have been made to synthesize superconducting antimonates, but with limited success. It was reported that partially doped BaPb_0.75_Sb_0.25_O_3_ becomes superconducting^32^, but its *T*_c_ is substantially decreased compared to that of BPBO. However, superconducting perovskite antimonates, with only antimony occupying the octahedral sites of perovskites, have yet to be experimentally reported, to the best of our knowledge. This is probably because the strongly covalent Sb–O bond is known to hamper forming 180 degree Sb–O–Sb bonds^33^, and as a consequence, no perovskite antimonates have been realized to date except a highly distorted insulating NaSbO_3_ (ref. ^34^). Here, we report superconducting antimonates Ba_1−*x*_K_*x*_SbO_3_ (BKSO), which we were able to stabilize via high-pressure high-temperature synthesis routes, enabling clarification of possible driving mechanisms for CDW and superconductivity in the compounds by comparing their properties with the sibling compound, BKBO.

**Fig. 1:**
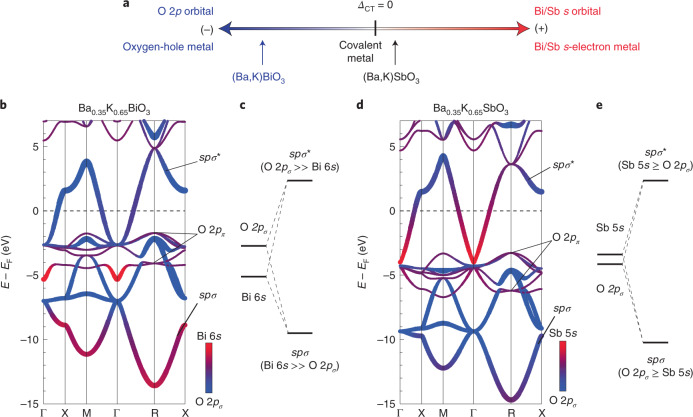
Inverted charge transfer energy in between BKSO and BKBO. **a**, A schematic diagram of different regimes of metallicity in BKBO and BKSO. When charge transfer energy *Δ*_CT_ is positive (negative), Bi or Sb *s* electrons (oxygen holes) are predominant. **b**, The fat-band representation of the electronic band structure of BKBO at *x* = 0.65 calculated via hybrid DFT. The thickness is proportional to the sum of O 2*p*_*σ*_ and Bi 6*s* contributions, and the colours represent their ratio, with predominant O 2*p*_*σ*_ character shown by blue and predominant Bi 6*s* character by red. The plot shows the predominant O 2*p*_*σ*_ (Bi 6*s*) character in the spσ* (*spσ*) band. A band with predominant Bi 6*s* character at Γ at −5.4 eV is formed by non-bonding Bi 6*s* states (Extended Data Fig. 2 for the O 2*p*_*π*_ contribution). *E*_F_, Fermi energy. **c**, The molecular-orbital diagram of BKBO derived from **b**. The Bi 6*s* orbital energy is markedly lower than the O 2*p* energy, consistent with negative *Δ*_CT_. Therefore, BKBO is located in the scheme of the oxygen-hole metal^20,21^, as illustrated in **a**. **d**, The fat-band representation of the electronic band structure of BKSO at *x* = 0.65 calculated via hybrid DFT. Sb 5*s* and oxygen 2*p* are found to be highly mixed in both *spσ* and spσ* bands. The much enhanced Sb 5*s* character in the spσ* band is clear compared to that of Bi 6*s* in BKBO. The non-bonding Sb 5*s* states at Γ are at −4.0 eV (Extended Data Fig. 2 for the O 2*p*_*π*_ contribution). **e**, The molecular-orbital diagram for BKSO derived from **d**. The Sb 5*s* orbital energy is marginally higher than the O 2*p* energy, indicating that *Δ*_CT_ is slightly positive while being close to zero (*Δ*_CT_ ≳ 0). Thus, BKSO is located in the region of the Bi/Sb *s*-orbital metal while critically close to the covalency limit in **a**.

**Extended Data Fig. 1:**
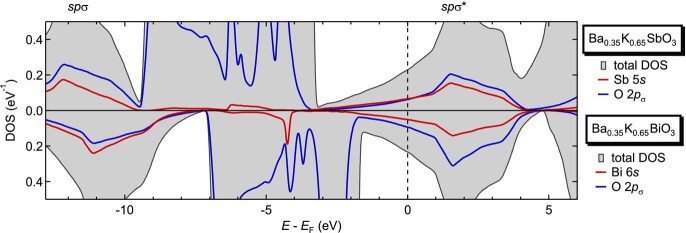
Electronic density of states of Ba_0.35_K_0.65_SbO_3_ and Ba_0.35_K_0.65_BiO_3_. The results are consistent with those suggested from the molecular-orbital diagrams (Figs. 1c and 1e); Ba_0.35_K_0.65_BiO_3_ with negative *Δ*_CT_ shows predominant Bi 6*s* and O 2*p*_*σ*_ characters in the *spσ* and spσ* bands, respectively. The projected density of states (PDOS) of O 2*p*_*σ*_ at the Fermi level is larger than that of Bi 6*s* [PDOS (Bi 6*s*) / PDOS (O 2*p*_*σ*_) ≅ 0.552]. On the contrary, for Ba_0.35_K_0.65_SbO_3_ with *Δ*_CT_ slightly positive while close to zero, the ratio between Sb 5*s* and O 2*p*_*σ*_ PDOS is not so different between the *spσ* and spσ* bands, and very close to unity at the Fermi level [PDOS(Sb 5*s*) / PDOS (O 2*p*_*σ*_) ≅ 1.06)]. Thus, metal *s* character with respect to O 2*p*_*σ*_ at the Fermi level is stronger in Ba_0.35_K_0.65_SbO_3_ than Ba_0.35_K_0.65_BiO_3_. Total DOS at the Fermi level shows almost no difference between the two compounds (Ba_0.35_K_0.65_SbO_3_: 0.223 states/eV, Ba_0.35_K_0.65_BiO_3_: 0.227 states/eV).

**Extended Data Fig. 2:**
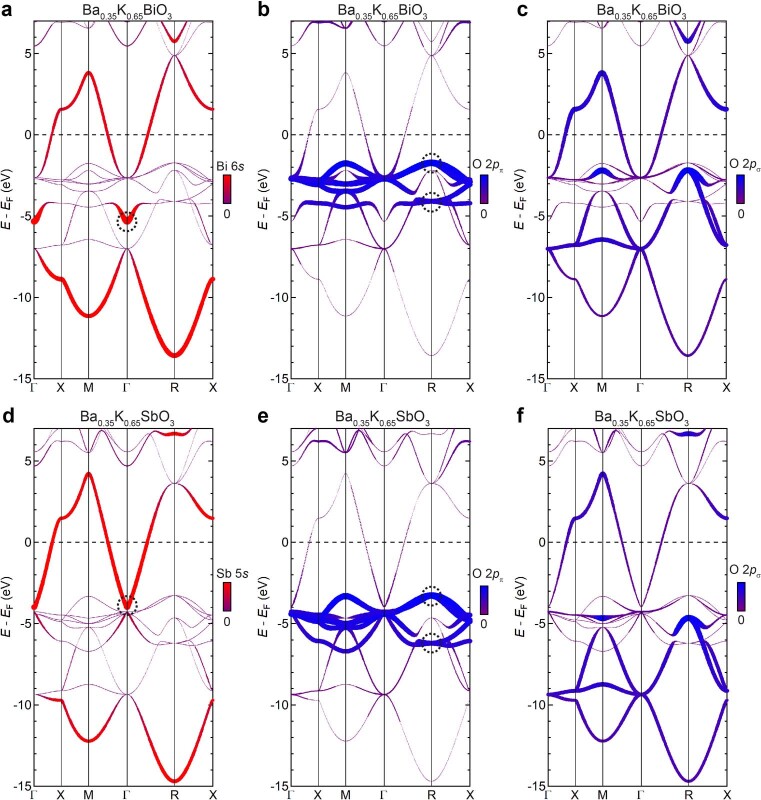
Metal *s* and oxygen 2*p* characters of Ba_0.35_K_0.65_SbO_3_ and Ba_0.35_K_0.65_BiO_3_. The fat-band representations of the band structure of Ba_0.35_K_0.65_BiO_3_ calculated via the hybrid-DFT showing **a**, Bi 6*s*, **b**, O 2*p*_*π*_, and **c**, O 2*p*_*σ*_ orbital characters. The black dashed circle in **a** denotes a band at Γ at which Bi 6*s* (*a*_1g_ symmetry) do not hybridize with any O 2*p* states in the cubic structure. The energy of this band can be used to estimate the on-site energy of Bi 6*s*^31,34,35^, if one neglects weak Bi 6*s*–O 2*s* hybridization. The two black dashed circles in **b** denote triply degenerate bands at R at which the O 2*p*_*π*_ states have the *t*_2g_ and *t*_1g_ symmetries, respectively, and do not hybridize with Bi 6*s* or 6*p* states. The on-site energy of O 2*p*_*π*_ can be reasonably approximated by averaging the energies of these two bands, which cancels out the effect of O 2*p*–O 2*p* hybridization. The fat-band representations of the electronic band structure of Ba_0.35_K_0.65_SbO_3_ calculated via the hybrid-DFT showing **d**, Sb 5*s*, **e**, O 2*p*_*π*_, and **f**, O 2*p*_*σ*_ orbital characters. The black dashed circles in **d** and **e** denote the bands with Sb 5*s* (*a*_1g_) and O 2*p*_*π*_ *t*_2g_ and *t*_1g_ characters as in the case of Ba_0.35_K_0.65_BiO_3_.

To shed light on the effects that varying *Δ*_CT_ has on the electronic structure, the band structures of BKSO as well as BKBO are calculated via a hybrid density functional theory (DFT) method^10^, as shown in Fig. 1 (total and projected density of states (DOS) are in Extended Data Fig. 1). The calculations are conducted for primitive cubic structures without the breathing distortions, which were experimentally obtained at the potassium concentration *x* = 0.65. The band structure of BKBO in Fig. 1b comprises the spσ* band around the Fermi level and the *spσ* band below –7 eV, which lie above and below the non-bonding flat O 2*p*_*π*_ bands (around –2 and –4 eV), respectively. The band structure of BKSO in Fig. 1d shares these common overall features, but the locations of the O 2*p*_*π*_ bands are pushed down appreciably, demonstrating the elevated Sb 5*s* orbital energy relative to the Bi 6*s*, as pointed out previously^29–31^. In Fig. 1b,d, the sum of the O 2*p*_*σ*_ and Bi-6*s*/Sb-5*s* contributions and their composition are indicated by the thickness and colour; blue denotes predominant O 2*p*_*σ*_ character and red denotes predominant Bi-6*s*/Sb-5*s* character (the O 2*p*_*π*_ contribution is in Extended Data Fig. 2). The analysis of the orbital composition indeed demonstrates that *Δ*_CT_ for BKSO is almost zero and slightly positive, in contrast to BKBO with negative *Δ*_CT_, as schematically shown in Fig. 1a. In BKBO, the red region with predominant Bi 6*s* character can be identified at the Γ point. Its energy, *E* = − 5.4 eV, is a good measure of the Bi 6*s* orbital energy, as the Bi 6*s* state does not hybridize with O 2*p* states at Γ in the cubic symmetry^31,34,35^. Clearly, the energy of the Bi 6*s* state is below the average energy of the flat O 2*p*_*π*_ bands, *E* = –2.9 eV (Extended Data Fig. 2 and Supplementary Table 1). By sharp contrast, the location of the red region at the Γ point in BKSO, a measure of the Sb 5*s* orbital energy, is at *E* = –4.0 eV, which is only slightly above the average energy of the O 2*p*_*π*_ bands, *E* = –4.8 eV. The clear contrast of predominant orbital character between the lower *spσ* and higher spσ* bands, the Bi 6*s* (red) and O 2*p*_*σ*_ (blue) characters, respectively, is evident for BKBO in Fig. 1b, indicating that the orbital energy of O 2*p*_*σ*_ is higher than that of Bi 6*s*, namely, *Δ*_CT_ < 0. In BKSO (Fig. 1d), the red/blue contrast is reversed, with more O 2*p*_*σ*_ character (blue) in the *spσ* band and more Sb 5*s* character (red) in the spσ* band, meaning *Δ*_CT_ ≥ 0. The more covalent character of BKSO, closer to the covalent limit *Δ*_CT_ = 0 compared to BKBO, can be seen as the strongly reduced contrast between the *spσ* and spσ* bands in the ratio of Sb 5*s* and O 2*p*_*σ*_ contributions to the DOS in Extended Data Fig. 1. We note that the previous estimates of *Δ*_CT_ in a hypothetical BSO based on a tight-binding fitting gave the sign of *Δ*_CT_ as either positive^29^ or negative^31^, depending on the model used. Incidentally, a fitting of the present band calculation based on Wannierization (Supplementary Fig. 1 and Supplementary Table 2) gives a positive value. The estimates of *Δ*_CT_ are quite useful in visualizing material trends within the same model, but are subject to model-dependent uncertainties in quantitative comparison across different models, as each model uses limited and different varied orbital bases and hopping terms to obtain ‘effective’ orbital energies and transfers. Here, we rely only on the qualitative arguments based on the orbital composition of the band structure to position BKSO in Fig. 1a.

**Fig. 2:**
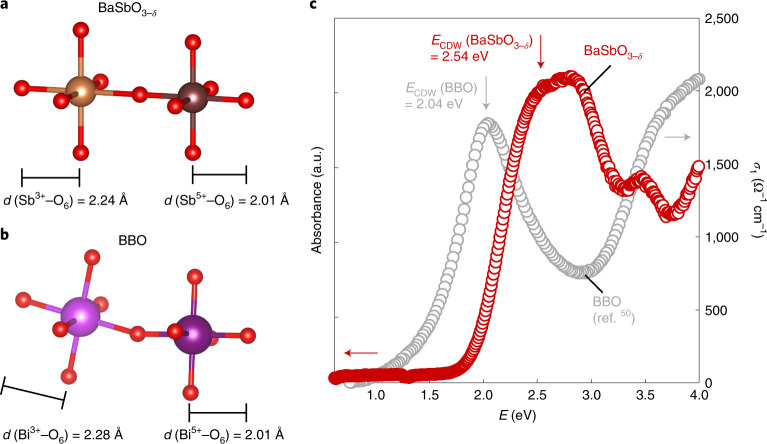
Three-dimensional CDW order in undoped BaSbO_3−*δ*_. **a**,**b**, Schematic diagrams of expanded and contracted octahedra in BaSbO_3−*δ*_ (**a**) and BBO (**b**). *d* denotes bond length between metal and oxygen ions. From the neutron diffraction investigations (Supplementary Fig. 2), two distinct Sb–O bond lengths are estimated to be 2.24(1) and 2.01(1) Å, respectively (Supplementary Table 3 for detailed structural parameters). The difference between the two bond lengths is found to be larger than that of BBO (ref. ^4^). **c**, Optical absorbance of BaSbO_3−*δ*_ at 300 K shows a wide bandgap *E*_CDW_ of 2.54 eV caused by the formation of the CDW order. By comparison, the optical conductivity (*σ*_1_) of BBO (ref. ^50^) is plotted as a reference, indicating the CDW gap of BaSbO_3−*δ*_ is larger than that of BBO.

Having clarified the inverted *Δ*_CT_ of BKSO as compared to that of BKBO, we next reveal how the inversion modifies physical properties of the materials. Polycrystalline samples of BKSO with potassium content *x* from 0 to 0.75 were synthesized under a high-pressure, high-temperature condition of 12 GPa and 1,300 °C (Methods for details of sample synthesis and characterization). The parent compound BaSbO_3−*δ*_ is found to be a robust insulator with a large CDW gap. Rietveld refinement of neutron powder diffraction data (Supplementary Fig. 2a) confirms face-centred cubic symmetry (space group *Fm*$\bar 3$*m*) with the breathing distortion, and furthermore reveals two distinct Sb–O bond lengths, 2.24(1) and 2.01(1) Å (Fig. 2a). Surprisingly, the difference between the two bond lengths Δ*d* = 0.23 Å is larger than that of BBO (Δ*d* = 2.28 − 2.11 = 0.16 Å, Fig. 2b)^4^. The observation of the breathing distortion establishes the commensurate CDW order from the structural point of view, which results in a bandgap in the material. Using diffusive reflectance spectroscopy, the bandgap in BSO is determined to be 2.54 eV (Fig. 2c). The value is appreciably larger than that in BBO (2.02 eV)^36^, consistent with the larger bond-length disproportionation observed in the neutron diffraction.

**Fig. 3:**
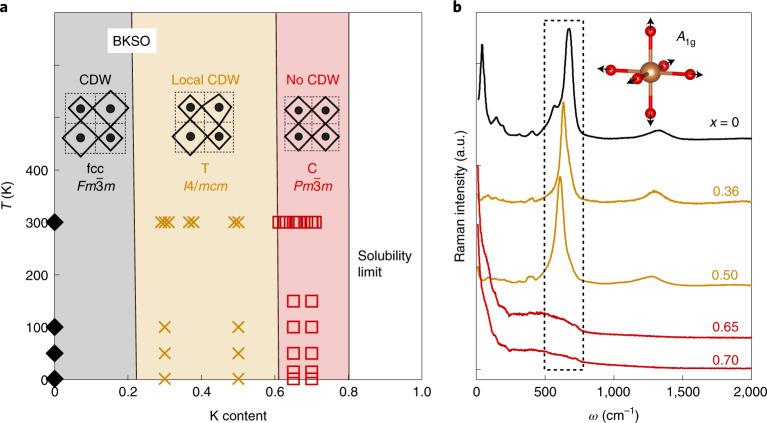
Suppression of the CDW order via potassium doping. **a**, The structural phase diagram of BKSO based on neutron (Supplementary Fig. 2) and X-ray (Supplementary Fig. 3) diffraction data. Black diamonds, tan crosses and red squares represent the face-centred cubic (fcc, *Fm*$\bar 3$*m*), tetragonal (T, *I*4/*mcm*) and primitive cubic (C, *Pm*$\bar 3$*m*) phases, respectively. The insets depict the local atomic structure of each phase, which shows transitions of the CDW order from the commensurate long range (*Fm*$\bar 3$*m*) to short range (*I*4/*mcm*), and finally to complete suppression (*Pm*$\bar 3$*m*). **b**, Raman spectra of BKSO measured with the excitation wavelength of 632 nm at 300 K. *ω* denotes the Raman shift in the unit of wavenumber. As highlighted in the dashed box, the breathing-mode phonon peak observed in the *Fm*$\bar 3$*m* and *I*4/*mcm* phases disappears in the *Pm*$\bar 3$*m* phase (*x* ≥ 0.65), confirming the CDW order is completely suppressed. The inset shows a schematic picture of the breathing-mode phonon.

The CDW order of the antimonate can be suppressed by substituting Ba with K up to 65%. X-ray and neutron powder diffraction measurements enable us to map out the structural phase diagram of BKSO, as shown in Fig. 3a (detailed refinement profiles and refined parameters are in Supplementary Figs. 2 and 3 and Supplementary Table 3). As *x* increases, two structural transitions are found, namely, from *Fm*$\bar 3$*m* to a tetragonal *I*4/*mcm* at *x* ≈ 0.3, and from *I*4/*mcm* to a primitive cubic *Pm*$\bar 3$*m* phase at *x* ≈ 0.65. This structural phase diagram is qualitatively similar to that of BKBO^37^, in which the CDW order is sequentially suppressed from the long range (*I*2/*m*) to short range (*Ibmm*) and then finally disappears (*Pm*$\bar 3$*m*). The structural transitions trigger drastic successive changes in Raman scattering, as shown in Fig. 3b. The undoped compound shows a pronounced peak at 672 cm^−1^, which corresponds to the breathing-mode phonon, that is, the symmetric movement of oxygen ions with *A*_1g_ symmetry, and is also known to be crucial to superconductivity in BKBO^38^. The phonon peak is first marginally softened as *x* increases, and next its amplitude completely vanishes for *x* ≥ 0.65. Because all the phonon modes become Raman-inactive in the *Pm*$\bar 3$*m* phase^39^, the vanishing breathing-mode peak confirms that the crystal symmetry above *x* = 0.65 is indeed *Pm*$\bar 3$*m* with the ideal cubic perovskite structure without any distortion; that is, the CDW order is completely suppressed. Interestingly, the critical potassium concentration *x*_IMT_ at which the structure symmetry becomes *Pm*$\bar 3$*m* is larger in BKSO (*x*_IMT_ ≈ 0.65) than in BKBO (*x*_IMT_ ≈ 0.35). The larger *x*_IMT_ is plausibly related to the bigger CDW gap, which may necessitate more holes for its suppression in the antimonates.

**Fig. 4:**
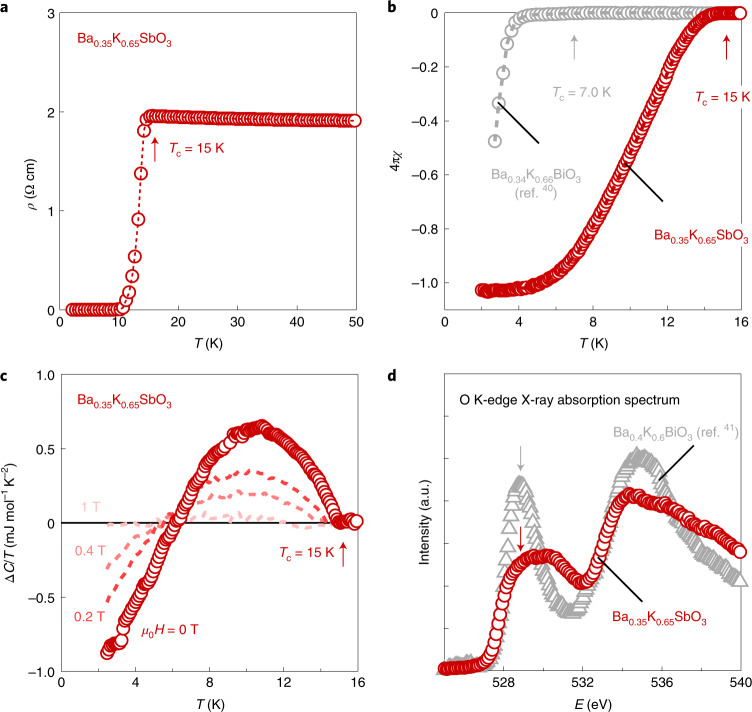
Superconductivity and weakened oxygen-hole character in Ba_0.35_K_0.65_SbO_3_. **a**, Superconducting transition observed in the resistivity (*ρ*) of optimally doped antimonate (*x* = 0.65). *T* denotes temperature. The superconducting transition temperature *T*_c_, defined by the clear onset of the transition, is ~15 K. **b**, The superconducting transition of the same sample is observed in zero-field-cooled magnetic susceptibility (*χ*) measured at *μ*_0_*H* = 0.001 T (red), in comparison with that of Ba_0.34_K_0.66_BiO_3_ (grey)^40^. *﻿H* is an applied magnetic field, and *μ*_0_ is the vacuum permeability. The diamagnetic volume fraction is near 100%, indicating bulk superconductivity. Here, *T*_c_ is 15 K, defined as a temperature where the volume fraction started increasing by 0.1%. **c**, The superconducting transition of the same sample observed in the specific heat. Δ*C* denotes the difference between specific heats (*C*) under each field and 14 T. *T*_c_ is estimated to 15 K from the clear onset of jump, which can be suppressed by applying a field of 1 T. The observed jump is broadened, perhaps indicating sample inhomogeneity from the high-pressure synthesis. **d**, Oxygen K-edge X-ray absorption spectrum of Ba_0.35_K_0.65_SbO_3_ (red open circles) at 300 K, plotted together with that of Ba_0.4_K_0.6_BiO_3_ (ref. ^41^; grey open triangles). The intensity of each spectrum is normalized by that at a high energy ~550 eV above the edge. The arrows indicate the prepeak structure originating from oxygen 2*p* holes in the spσ* band. The suppression of the prepeak intensity in the antimonate indicates the decrease of oxygen holes compared to the bismuthate.

The BKSO samples with *x* ≧ 0.65 and *Pm*$\bar 3$*m* symmetry shows bulk superconductivity with a maximum *T*_c_ = 15 K at *x* = 0.65 (Fig. 4). The superconducting transition of the optimally doped sample (*x* = 0.65) is clearly identified at *T*_c_ = 15 K in resistivity (Fig. 4a). In the magnetic susceptibility (Fig. 4b), a diamagnetic signal corresponding to a superconducting volume fraction near 100% is observed below *T*_c_ = 15 K. The specific heat shows a jump with an onset at 15 K due to the superconducting transition, which is suppressed by applying a magnetic field of 1 T (Fig. 4c). The magnitude of the jump is on the order of the linear specific heat coefficient *γ* (0.924 mJ mol^−1^ K^−2^; Supplementary Fig. 4), confirming the bulk nature of superconductivity. The maximum *T*_c_ = 15 K of BKSO at *x* = 0.65 is found to be lower than that of BKBO at *x* = 0.4, *T*_c_ ≈ 30 K, but at comparable potassium concentrations, is higher than that of BKBO (7.0 K at *x* = 0.66 (ref. ^40^)) by more than a factor of two, as seen in Fig. 4b.

**Extended Data Fig. 3:**
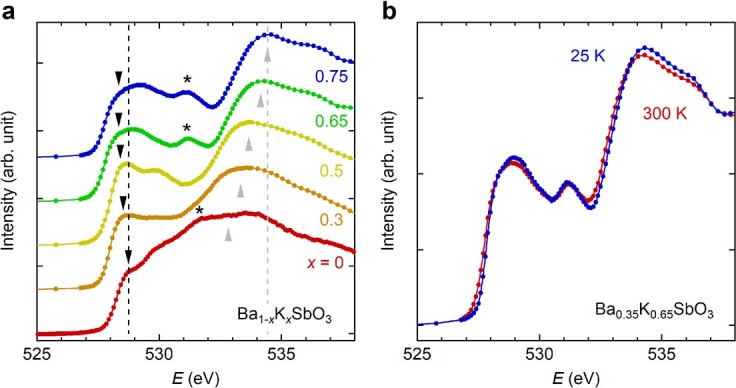
The oxygen K-edge X-ray absorption spectra of Ba_1-*x*_K_*x*_SbO_3_ (0 ≤ *x* ≤ 0.75). **a**, The spectra of the BKSO samples with various K contents were measured at 300 K in the total fluorescence yield mode. All the samples show the pre-peak structure (black arrows) analogous to the Ba_0.35_K_0.65_SbO_3_ sample depicted in Fig. 4d. This indicates a strong admixture of Sb 5*s* with O 2*p* throughout the entire *x* range, but the intensity of the pre-peak is appreciably reduced compared with that of BKBO at comparable *x* values^41,42,55^. The position of the pre-peak shifts to a lower energy with increase of *x*, which can be understood as the suppression of the CDW gap by doping holes^41,55^. The high-energy peak (grey arrows) shifts to a higher energy, which may be associated with the Ba 5*d* and K 3*d* states that change upon the chemical substitution^41^. For the *x* = 0, 0.65, and 0.75 samples, the extra peak around 531 eV (black asterisks) likely originates from a degraded surface, because of its pronounced intensity in the surface-sensitive total electron yield mode data (not shown). Further quantitative analysis has so far been limited at present, as the Sb M_5_-edge gives rise to an additional structure around the pre-peak because its energy is so close to that of the oxygen K-edge^56^. **b**, The spectra of the *x* = 0.65 sample at 300 and 25 K, showing no apparent change within the temperature range investigated. We note that the *x* = 0.65 sample shown here is different from that in Fig. 4d.

Reduced oxygen-hole character in BKSO is revealed via X-ray absorption spectroscopy, consistent with its positive *Δ*_CT_. The oxygen K-edge, which probes unoccupied oxygen 2*p* states, is measured for the optimally doped BKSO and compared with a BKBO reference^41^ at similar *x* in Fig. 4d. The BKBO (*x* = 0.6) exhibits a pronounced prepeak structure at *E* = 528.8 eV, indicating predominant oxygen holes in the conduction band. The BKSO (*x* = 0.65) sample shows a similar prepeak at the same energy, but its intensity is appreciably diminished, indicating the reduction of the oxygen-hole density. The suppression of the prepeak intensity in BKSO compared with BKBO^41,42^ is observed not only around *x* = 0.65, but also in all the available *x* range including *x* = 0 (BSO; Extended Data Fig. 3). We also note that a clear signature of charge disproportionation of Sb^3+^ and Sb^5+^ has been observed very recently in ^121^Sb Mössbauer spectroscopy in BSO^43^. No such clear spectroscopic signature of Bi^3+^ and Bi^5+^ has been reported in BBO which can be attributed to the predominant oxygen holes^24–26^. The stark contrast between BKSO and BKBO supports the strong metal *s* character and the reduced O 2*p* character of the spσ* band and hence the positive sign of *Δ*_CT_ in BKSO.

**Fig. 5:**
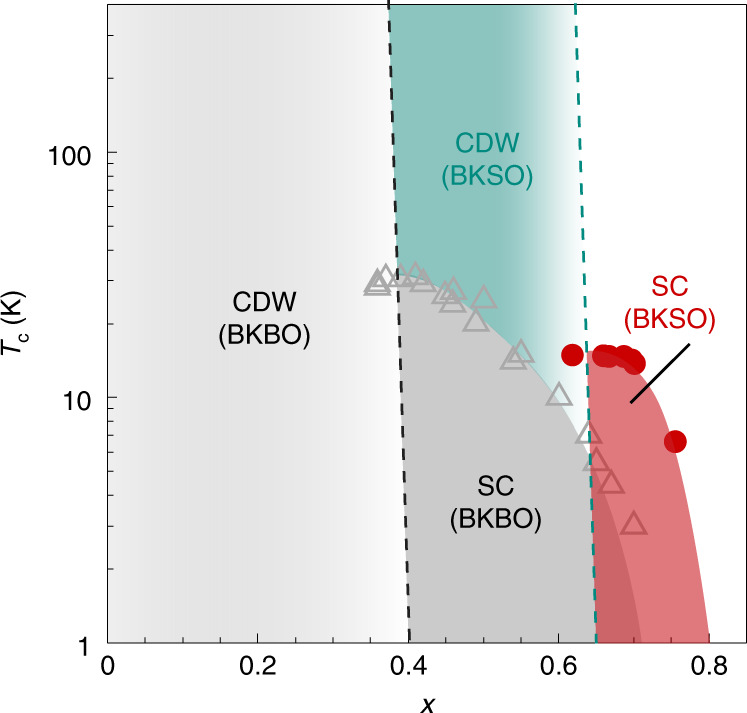
Phase diagram of BKSO and BKBO. Red circles are the *T*_c_ values of superconductivity (SC) in the antimonates with the same definition as in Fig. 4, and grey triangles are *T*_c_ of BKBO (refs. ^40,51^). *T*_c_ values of the antimonates show a half-dome shape (red region), which is similar to that of the bismuthates (dark grey). The crucial difference is that the CDW order in the bismuthates (light grey region) is suppressed at *x* = 0.4, whereas that of the antimonates (green region) continues to exist up to *x* = 0.65.

A phase diagram of BKSO, compiled from these results, offers a comprehensive view of the interplay between the CDW order and superconductivity in main-group oxide superconductors (Fig. 5). First, a common tendency in the phase diagram can be found in both compounds; as the CDW insulating phase is suppressed, a half-dome of superconductivity arises with *T*_c_ maximized at the border of the insulator-to-metal transition and gradually decreasing with *x* increasing. Nevertheless, a crucial difference between the two compounds is that the suppression of the CDW phase occurs at higher *x* in BKSO, possibly related to its larger CDW gap. As BKSO shows a higher *T*_c_ at *x* ≥ 0.65, its *T*_c_ could exceed that of BKBO if it were possible to stabilize metallic BKSO at lower *x* and if the same trend of *T*_c_ based on *x* held. In reality, this has so far been prohibited by the strong CDW instability in BKSO, setting a limit on enhancing the superconductivity further.

The superconducting *T*_c_ that is more than doubled in BKSO at *x* = 0.65 suggests a mechanism associated with strong metal–oxygen covalency. The electronic structure in Fig. 1e and Extended Data Fig. 1 points to the close proximity of the orbital energies of Sb 5*s* and O 2*p*, *Δ*_CT_ ≈ 0, indicating the strong covalency in BKSO. This leads to the Sb–O bonds being stiffer than the Bi–O bonds, and the relevant phonon energies of BKSO being higher than those of BKBO, as supported by the following experiments. First, the Debye temperature of the optimally doped BKSO was found to be 535 K (Supplementary Fig. 4), which is greater than that of BKBO, typically ~330 K (ref. ^44^). Second, the breathing-mode phonon in the parent compounds showed its frequency increased by about 19% in BSO (672 cm^−1^), as compared to BBO^39^ (565 cm^−1^; Supplementary Fig. 5). The increase of the phonon frequencies can be a factor in increasing *T*_c_ but alone cannot fully account for the more than twofold enhancement of *T*_c_ in BKSO at *x* = 0.65 in terms of the Macmillan’s formula for superconducting *T*_c_. This implies that the increase of the electron–phonon coupling, possibly linked with the enhanced covalency, must be incorporated in the enhancement of superconductivity as well (Supplementary Note 1 for more detailed discussion). The stronger CDW instability in BKSO could also be related to such an enhanced electron–phonon coupling, which is reminiscent of covalent superconductors in which too large electron–phonon coupling sometimes leads to lattice instability instead of higher *T*_c_ (refs. ^45,46^).

Finally, yet importantly, our study affords insight into the role of negative *Δ*_CT_ and predominant oxygen holes. On one hand, it is evident from the results that BKSO, which has slightly positive *Δ*_CT_, demonstrates both CDW insulating and superconducting phases, analogous to BKBO. Therefore, the emergence of the two phases is not necessarily dependent on the sign of *Δ*_CT_. In addition, at *x* ≥ 0.65, BKSO shows even higher *T*_c_ than BKBO, in spite of its decreased oxygen-hole character. Hence, it could be inferred that predominant oxygen holes may not be a necessary condition for enhancement of superconductivity. On the other hand, BKBO, which has more oxygen holes, shows a smaller amplitude of the CDW order than that in BKSO, which results in a smaller *x*_IMT_ as well. Since the electronic DOS at the Fermi level as well as the electron–phonon interaction become larger with decreasing *x* (ref. ^14^), a smaller *x*_IMT_ would lead to an increased *T*_c_. Therefore, the weakened CDW order in BKBO, likely by predominant oxygen holes, could be vital to show a higher *T*_c_. In this respect, predominant oxygen holes may be a sufficient condition for a higher *T*_c_.

We have reported superconducting perovskite antimonates with a maximum *T*_c_ of 15 K. The modification of *Δ*_CT_ via the substitution of Bi with Sb has allowed us to address long-standing questions as to the different roles of metal and oxygen ions for the CDW and superconductivity in the main-group oxide superconductors. Furthermore, these results provide fascinating possibilities for approaching novel regimes in the future. For example, it would be intriguing to modify *Δ*_CT_ either to be more positive or negative by utilizing appropriate elements at the octahedral site: As and Sn would give an on-site energy of a metal *s* level higher than Sb, and thus more positive *Δ*_CT_ value than antimonates. Assuming these compounds can be stabilized, they would show more prominent effects of the cations, providing ideal model systems to examine the negative *U* model and the effects of valence fluctuations^18^. On the other hand, Te (ref. ^47^) and I (ref. ^48^) would give an on-site energy of a metal *s* level lower than Sb, and perhaps comparable or even lower than Bi. Thus, their *Δ*_CT_ values would be more negative than those of the antimonates and possibly bismuthates, providing additional model systems to comprehensively examine the effect of oxygen holes^20^. We also note that charge- or bond-disproportionated CDW states are not limited to oxides but extend to other families, including perovskite halides CsTlCl_3_ or CsTlF_3_ (ref. ^49^).

## Methods

### Sample synthesis and characterization

Polycrystalline samples of BKSO (*x* = 0.0, 0.36, 0.50, 0.65 and 0.75) were fabricated using a high-pressure high-temperature synthesis technique with a Walker-type multi-anvil module. Precursors of BaO_2_ (95%, Acros Organics), KO_2_ (96.5%, Alfa Aesar), Sb (99.999%, ChemPur) and Sb_2_O_3_ (99.999%, Aldrich) were mixed in stoichiometric ratios and transferred into a Pt capsule. The samples were obtained from high-pressure high-temperature treatment at 12 GPa and 1,300 °C for an hour, followed by quenching to room temperature and slow decompression. A dense pellet of the samples was recovered from the Pt capsule, after which its outer face was polished using a diamond file. Powder X-ray and neutron diffraction data were collected in the Debye–Scherrer geometry using a Mo Kα_1_ source and a time-of-flight neutron source, respectively, with the instrument WISH at ISIS. The structural refinements based on neutron and X-ray diffraction patterns, shown in Supplementary Figs. 2 and 3, indicate that the samples consist of an almost pure BKSO perovskite phase with only a very minor trace of BaSbO_2.5_ for *x* = 0 and KSbO_3_ for *x* ≠ 0 as the impurity phases. The X-ray refinements reveal no clear signature of cation non-stoichiometry and the K contents, *x*, are in good agreement with the nominal values for all the samples. The neutron refinement suggests an oxygen deficiency on the order of a few percent only for BaSbO_3−*δ*_ (*x* = 0). The oxygen deficiency in BaSbO_3−*δ*_ is not large as compared with its sibling compound BaBiO_3−*δ*_, which is known to be easily reduced at relatively low temperatures and oxygen partial pressures^52^.

### Physical property measurements

Magnetic susceptibility was measured via a Quantum Design magnetic property measurement system, and resistivity and heat capacity were measured via a Quantum Design physical property measurement system. Optical absorbance was measured via diffuse reflectance spectroscopy at room temperature. X-ray absorption spectroscopy was measured in partial fluorescence yield mode using a silicon drift detector to select the O K-edge fluorescence at the Spherical Grating Monochromator beamline and in total fluorescence yield mode using a microchannel plate at the Resonant Elastic and Inelastic X-ray Scattering beamline of the Canadian Light Source.

### First-principles calculation

The band structures of BKSO and BKBO were calculated using the WIEN2k code^53^ with full hybrid functionals (YS-PBE0, similar to HSE06 (ref. ^54^)). We found that a 12 × 12 × 12 reciprocal-space *k* mesh is sufficient for the calculations well converged. *R*_MT_*K*_MAX_ was set to  7.0, where *R*_MT_ is the smallest atomic sphere radius and *K*_MAX_ is the maximal *k* in the plane wave expansion. The virtual crystal approximation was used to take into account the solid solution of Ba and K ions. The atomic structures reported from experiments were used.

## Online content

Any methods, additional references, Nature Research reporting summaries, source data, extended data, supplementary information, acknowledgements, peer review information; details of author contributions and competing interests; and statements of data and code availability are available at 10.1038/s41563-022-01203-7.

## Supplementary information

Supplementary Information Supplementary Note 1, Figs. 1–5 and Tables 1–3.

## Data Availability

The data that support the findings of this work are available from the corresponding authors upon reasonable request.
