# Supplementary material for: Superconductivity in (Ba,K)SbO3
Source: Nat Mater. 2022 Feb 28;21(6):627–33. doi: 10.1038/s41563-022-01203-7 (PMC9156407; doi:10.1038/s41563-022-01203-7)
Supplement: Supplementary file 1 — Supplementary Note 1, Figs. 1–5 and Tables 1–3. [file 41563_2022_1203_MOESM1_ESM.pdf]

nature portfolio

https://doi.org/10.1038/s41563-022-01203-7

Supplementary information

Superconductivity in (Ba,K)SbO_3

In the format provided by the authors and unedited

Supplementary Information for "Discovery of Superconductivity in (Ba,K)SbO_3"

Minu Kim[1], Graham M. McNally[1], Hun-Ho Kim[1], Mohamed Oudah[2], Alexandra S. Gibbs[3], Pascal Manuel[3], Robert J. Green[2,4], Ronny Sutarto[5], Tomohiro Takayama[1], Alexander Yaresko[1], Ulrich Wedig[1], Masahiko Isobe[1], Reinhard K. Kremer[1], D. A. Bonn[2], Bernhard Keimer[1], and Hidenori Takagi[1,6]

[1]Max Planck Institute for Solid State Research, Heisenbergstrasse 1, 70569 Stuttgart, Germany.

[2]Stewart Blusson Quantum Matter Institute, University of British Columbia, Vancouver, British Columbia V6T 1Z4, Canada.

[3]ISIS Facility, STFC Rutherford Appleton Laboratory, Harwell Science and Innovation Campus, Oxon OX11 0QX, United Kingdom.

[4]Department of Physics & Engineering Physics, University of Saskatchewan, Saskatoon, S7N 5E2, Canada.

[5]Canadian Light Source, University of Saskatchewan, Saskatoon, S7N 2V3, Canada.

[6]Department of Physics, University of Tokyo, Bunkyo-ku, Hongo 7-3-1, Tokyo 113-0033, Japan.

Supplementary Note 1. Implication of enhancement of T_c in $Ba_{1-x}K_xSbO_3$ (BKSO) as compared with $Ba_{1-x}K_xBiO_3$ (BKBO) at x = 0.65 within the Macmillan equation for T_c.

In the modified McMillan equation[1,2], T_c is given as $(\omega_{\log}/1.20)\exp[-1.04(1+\lambda)/\{\lambda-\mu^*(1+0.62\lambda)\}]$, where $\omega_{\log}$ is the characteristic logarithmic-averaged phonon frequency, λ is the electron-phonon coupling constant, and μ^* is the Coulomb pseudopotential parameter. As shown in Fig. S6, the Debye temperature Θ_D of BKSO is about 535 K, which is less than 50% larger than that of BKBO (Θ_D of BKBO is reported to be 330 K at x = 0.4[3], which is very likely larger at x = 0.65 because of the increased concentration of lighter potassium ions). The frequency of the breathing optical phonon mode is increased by 19 % in BSO compared with BBO. The associated increase of the prefactor $\omega_{\log}$ in the Macmillan equation is not large enough to explain the observed enhancement of T_c by more than a factor of two. Assuming μ^* does not change appreciably from BKBO to BKSO, λ must increase as well. λ is given as $N(E_F)\langle I^2\rangle/M\langle\omega^2\rangle$, where $N(E_F)$ is the DOS at the Fermi energy, $\langle I^2\rangle$ is the Fermi surface average of squared electron-phonon coupling interaction, M is the atomic mass, and $\langle\omega^2\rangle$ is the average of squared phonon frequency. $N(E_F)$ of BKSO and BKBO, estimated via the hybrid-DFT calculations, is nearly identical (see Extended Data Fig. 1). M should be dominated by the mass of oxygen in both compounds. With the increase of phonon frequency $\langle\omega^2\rangle$, the increase of λ in BKSO therefore implies an increased average electron-phonon interaction $\langle I^2\rangle$ as compared with BKBO.

Compound	E_s (eV)	$E_{p\ t2g}$ (eV)	$E_{p\ t1g}$ (eV)	$E_{p\pi} = (E_{p\ t2g} + E_{p\ t1g})/2$ (eV)	$\Delta_{CT} = E_s - E_{p\pi}$ (eV)
$Ba_{0.35}K_{0.65}BiO_3$	−5.37	−1.73	−4.11	−2.92	−2.45
$Ba_{0.35}K_{0.65}SbO_3$	−4.00	−3.29	−6.22	−4.76	0.757

Table S1 | Orbital energies of BKBO and BKSO at x = 0.65 directly estimated from the band calculations. E_s, $E_{p\ t2g}$, $E_{p\ t1g}$, $E_{p\pi}$ are the energies of M s, O 2p t_{2g}, O 2p t_{1g}, and O 2p_π orbitals, respectively. E_s of each compound is estimated from the non-bonding M s band at Γ in Extended Data Figs. 2a and 2d. $E_{p\pi}$ of each compound is estimated by averaging $E_{p\ t2g}$ and $E_{p\ t1g}$, the energies of the two triply degenerate O 2p_π bands in Extended Data Figs. 2b and 2e. We note that the on-site energy of O 2p_σ is unknown in this analysis, as there is no point in k-space at which hybridization between O 2p_σ and other orbitals can be neglected.

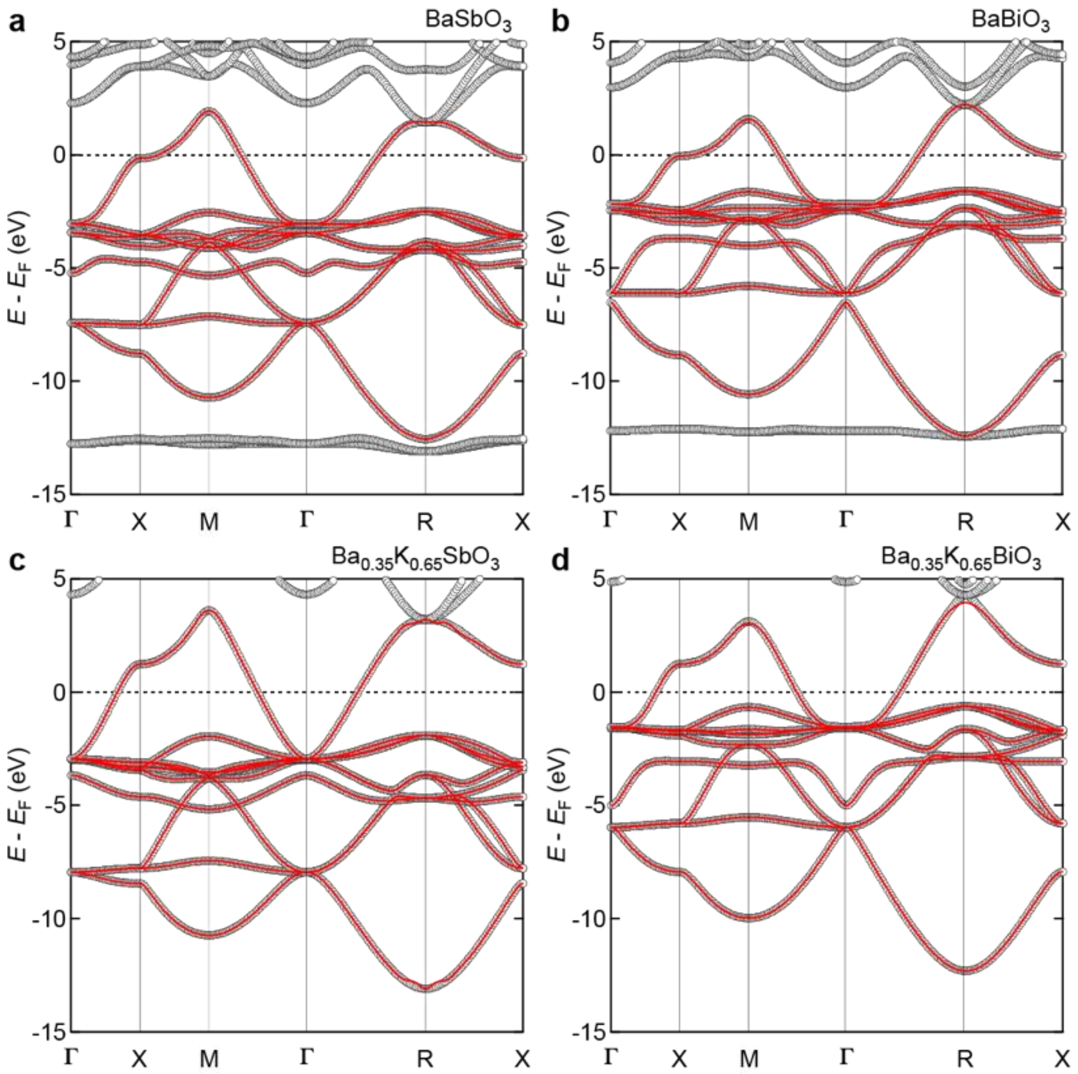

Figure S1 | Wannierization of BKSO and BKBO without and with potassium doping. For Wannierization, density functional theory calculations of the band structures of **a**, $BaSbO_3$, **b**, $BaBiO_3$, **c**, $Ba_{0.35}K_{0.65}SbO_3$, **d**, $Ba_{0.35}K_{0.65}BiO_3$ were performed with the WIEN2k code using the PBE GGA (generalized gradient approximation) to exchange correlation potential (black open circles). The other conditions are the same as those described in Methods. Wannierization using 7×7×7 *k* mesh and 10 Wannier projections (1 × metal *s* + 3 × oxygen $2p_x$, $2p_y$, $2p_z$) was performed to fit the DFT results (red curves).

Compound	E_s (eV)	$E_{p\sigma}$ (eV)	$E_{p\pi}$ (eV)	$t_{sp\sigma}$ (eV)	$\Delta_{CT} = E_s - (E_{p\sigma} + 2E_{p\pi})/3$ (eV)
$BaSbO_3$	−4.03	−5.67	−3.85	1.96	0.427
$BaBiO_3$	−5.30	−4.61	−2.80	2.00	−1.90
$Ba_{0.35}K_{0.65}SbO_3$	−2.59	−6.05	−3.58	2.13	1.81
$Ba_{0.35}K_{0.65}BiO_3$	−3.63	−4.21	−2.02	2.15	−0.890

Table S2 | Obtained parameters of BKSO and BKBO from Wannierization. E_s, $E_{p\sigma}$, $E_{p\pi}$ are the on-site energies of *M s* (*M* = Sb or Bi), O 2p_σ, and O 2p_π orbitals, respectively. $t_{sp\sigma}$ is the hopping parameter between *M s* and O 2p_σ, and Δ_{CT} is charge transfer energy defined as energy difference between the *M s* orbital and the average of O 2p_σ and O 2p_π. BSO and BKSO show positive Δ_{CT}, whereas BBO and BKBO show negative Δ_{CT}. The overall shift of Δ_{CT} to the positive side in the doped compounds, as compared to that of the undoped compound, may reflect the effect of increased *sp* hybridization, which is demonstrated as the increase of $t_{sp\sigma}$.

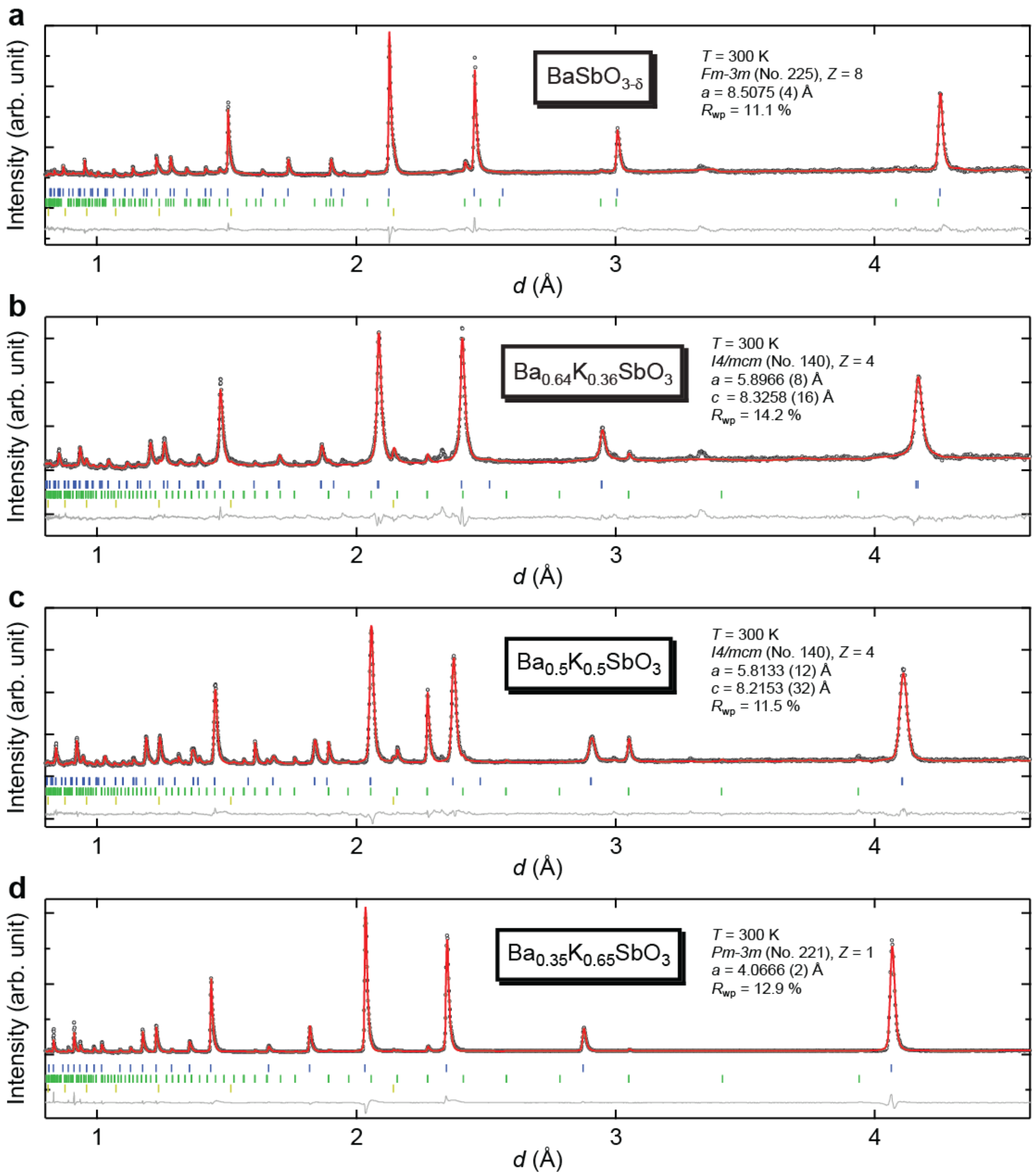

Figure S2 | Rietveld refinement profiles of diffraction patterns of BKSO samples based on powder neutron diffraction. Neutron diffraction patterns of **a**, $BaSbO_{3-\delta}$, **b**, $Ba_{0.64}K_{0.36}SbO_3$, **c**, $Ba_{0.5}K_{0.5}SbO_3$, **d**, $Ba_{0.35}K_{0.65}SbO_3$ were collected at the WISH diffractometer, ISIS facility. The black dots are experimental data, the red lines are the simulated intensity, and the blue, green, and yellow ticks are the Bragg reflections of $BaSbO_{3-\delta}$, impurity phases ($BaSbO_{2.5}$ in $BaSbO_{3-\delta}$, and

$KSbO_3$ in the other three samples) and a vanadium sample can, respectively. The grey lines are the difference between the experimental data and the simulation.

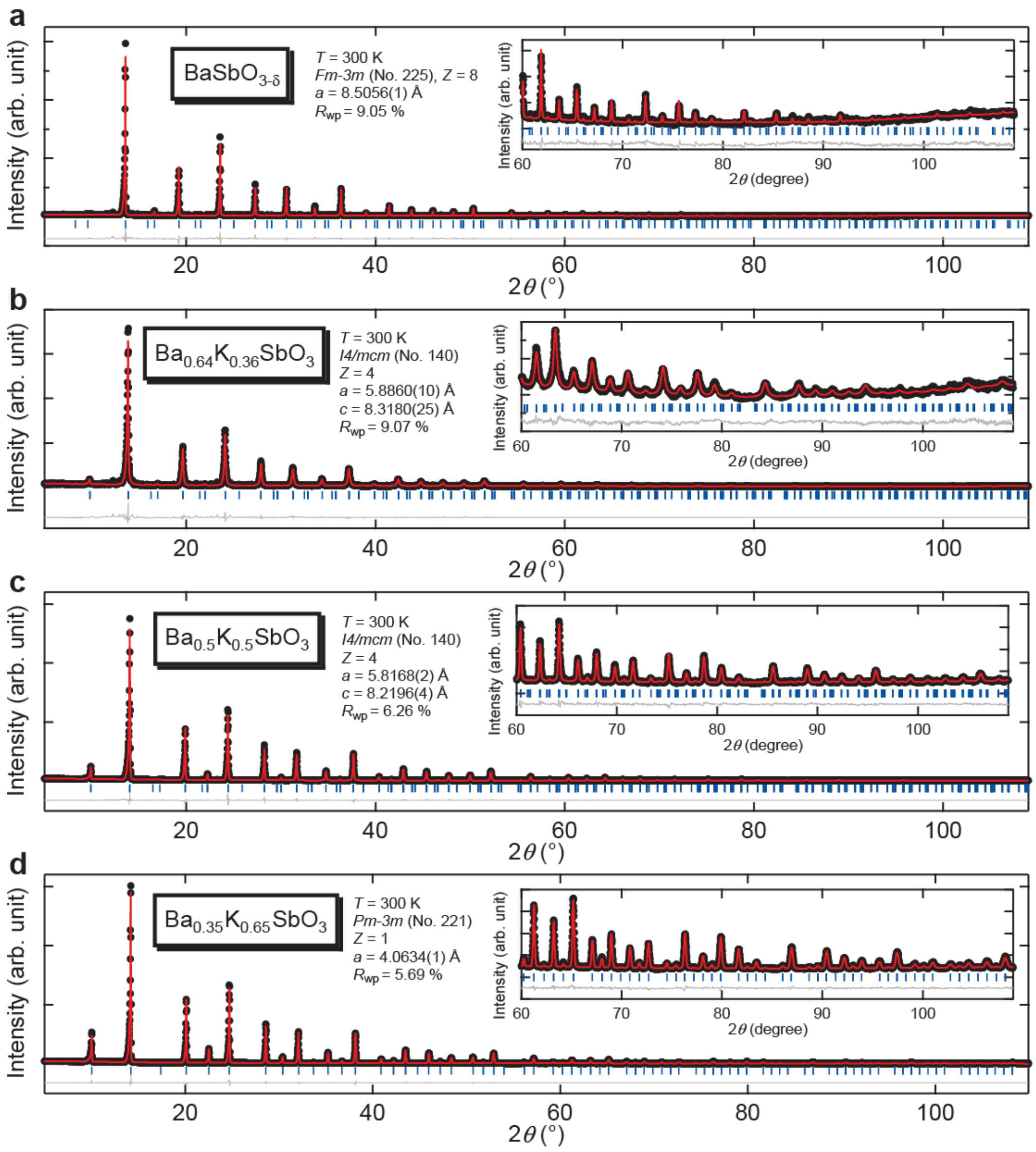

Figure S3 | Rietveld refinement profiles of diffraction patterns of BKSO samples based on powder X-ray diffraction. X-ray diffraction patterns of **a**, $BaSbO_{3-\delta}$, **b**, $Ba_{0.64}K_{0.36}SbO_3$, **c**, $Ba_{0.5}K_{0.5}SbO_3$, **d**, $Ba_{0.35}K_{0.65}SbO_3$ were collected in the Debye-Scherrer geometry with a Mo Kα_1 radiation at room temperature. Same notations as in Fig. S4 were used for plotting data.

Compound		$BaSbO_{3-\delta}$		$Ba_{0.64}K_{0.36}SbO_3$		$Ba_{0.50}K_{0.50}SbO_3$		$Ba_{0.35}K_{0.65}SbO_3$	
Diffraction data		XRD	ND	XRD	ND	XRD	ND	XRD	ND
Cell	Symmetry	$Fm\bar{3}m$	$Fm\bar{3}m$	$I4/mcm$	$I4/mcm$	$I4/mcm$	$I4/mcm$	$Pm\bar{3}m$	$Pm\bar{3}m$
	a / Å	8.5046(1)	8.5075(4)	5.8860(10)	5.8966(8)	5.8168(2)	5.8133(12)	4.0634(1)	4.0666(2)
	c / Å	-	-	8.3180(25)	8.3258(16)	8.2196(4)	8.2153(32)	-	-
Ba or K	x	0.25	0.25	0	0	0	0	0.5	0.5
	y	0.25	0.25	0.5	0.5	0.5	0.5	0.5	0.5
	z	0.25	0.25	0.75	0.75	0.75	0.75	0.5	0.5
	Occupancy	1	1	1	1	1	1	1	1
	Ba ratio, n	1	1	0.639(5)	0.64^{*}	0.504(2)	0.50^{*}	0.349(1)	0.35^{*}
	K ratio, $1-n$	0	0	0.361(5)	0.36^{*}	0.496(2)	0.50^{*}	0.651(1)	0.65^{*}
Sb1	$x = y = z$	0	0	0	0	0	0	0	0
	Occupancy	1	1	1	1	1	1	1	1
Sb2	$x = y = z$	0.5	0.5	-	-	-	-	-	-
	Occupancy	1	1	-	-	-	-	-	-
O1	x	0.2627^{**}	0.2627(3)	0	0	0	0	0	0
	y	0	0	0	0	0	0	0	0
	z	0	0	0.25	0.25	0.25	0.25	0.5	0.5
	Occupancy	0.971^{**}	0.971(1)	1^{**}	1	1^{**}	1	1	1
O2	x	-	-	0.243^{**}	0.243	0.247^{**}	0.247	-	-
	y	-	-	0.743^{**}	0.743	0.747^{**}	0.747	-	-
	z	-	-	0	0	0	0	-	-
	Occupancy	-	-	1^{**}	1	1^{**}	1	-	-
Ba or K, Biso / Å^2		1.60(6)	1.05(7)	0.47(5)	0.66(7)	0.572(11)	0.89(7)	0.450(10)	1.35(6)
Sb1, Biso / Å^2		2.05(10)	1.52(19)	0.99(5)	1.18(6)	0.407(8)	1.96(7)	0.320(6)	1.52(5)
Sb2, Biso / Å^2		0.87(6)	0.64(17)	-	-	-	-	-	-
O1, Biso / Å^2		5.3(2)	3.18(6)	$1.37(13)^{***}$	2.19(99)	$1.0(9)^{***}$	1.62(99)	0.69(3)	1.70(3)
O2, Biso / Å^2		-	-	$1.37(13)^{***}$	2.33(68)	$1.0(4)^{***}$	1.48(73)	-	-
R_{wp} / %		9.05	11.1	9.07	14.2	6.26	11.5	5.69	12.9
χ^2		9.73	3.19	9.23	7.49	2.32	5.46	1.29	37.4

Table S3 | Refined structural parameters of BKSO at 300 K from the powder X-ray diffraction (XRD) and neutron diffraction (ND) patterns. *Ba and K ratios in the neutron refinement are fixed with the values from the XRD refinement. **Atomic position and occupancy of oxygen in the XRD refinement are fixed to the values obtained from the neutron diffraction. ***Thermal parameter of O1 and O2 in the XRD refinement is fixed to be identical.

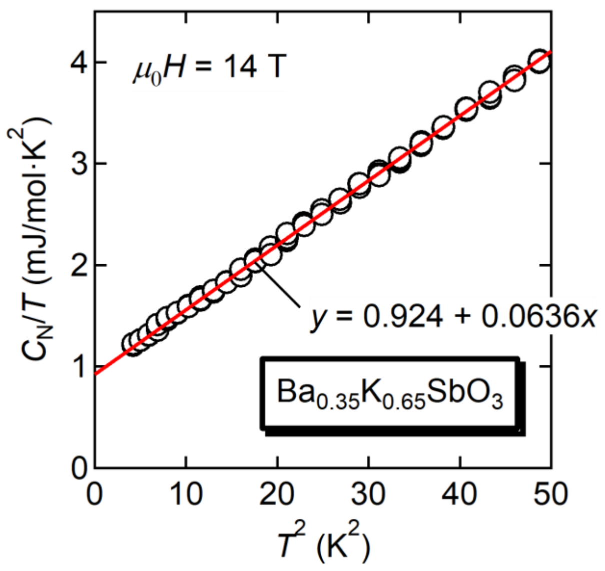

Figure S4 | Normal-state specific heat of the optimally doped BKSO. Normal-state specific heat C_N of $Ba_{0.35}K_{0.65}SbO_3$ was measured at a magnetic field of 14 T, which is far larger than the upper critical field (~1 T) of the compound. A fit to the data with $C_N (T) = \gamma T + \beta T^3$ yields γ = 0.924 mJ·mol^{-1}K^{-2} and β = 0.0636 mJ·mol^{-1}K^{-4}. From the value of β, the Debye temperature of 535 K was estimated.

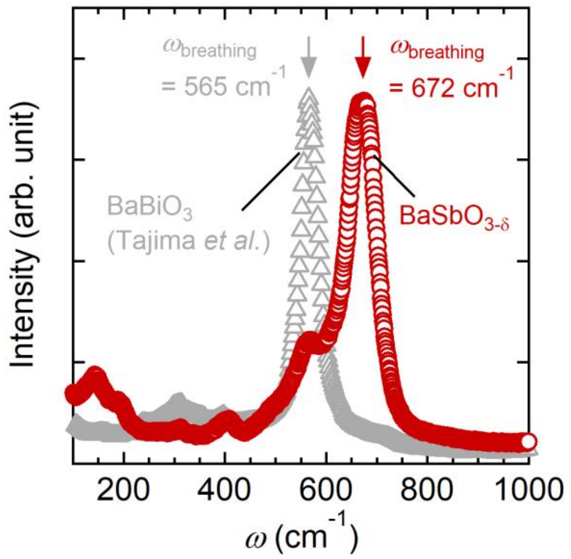

Figure S5 | Increased frequency of the breathing-mode phonon in the undoped antimonate as compared to the bismuthate. The frequency of breathing-mode phonon for $BaSbO_{3-\delta}$ (the red arrow) measured via Raman scattering shows a 19 % increase as compared to that of $BaBiO_3$[4] (the grey arrow), which we argue to reflect the enhanced metal-oxygen covalency of the antimonates.

References

1 McMillan, W. L. Transition temperature of strong-coupled superconductors. *Phys. Rev.* **167**, 331-344, doi:10.1103/PhysRev.167.331 (1968).

2 Allen, P. B. & Dynes, R. C. Transition temperature of strong-coupled superconductors reanalyzed. *Phys. Rev. B* **12**, 905-922, doi:10.1103/PhysRevB.12.905 (1975).

3 Woodfield, B. F., Wright, D. A., Fisher, R. A., Phillips, N. E. & Tang, H. Y. Superconducting-normal phase transition in $(Ba_{1-x}K_x)BiO_3$, $x = 0.40, 0.47$. *Phys. Rev. Lett.* **83**, 4622-4625, doi:10.1103/PhysRevLett.83.4622 (1999).

4 Tajima, S., Yoshida, M., Koshizuka, N., Sato, H. & Uchida, S. Rama-scattering study of the metal-insulator transition in $Ba_{1-x}K_xBiO_3$. *Phys. Rev. B* **46**, 1232-1235, doi:10.1103/PhysRevB.46.1232 (1992).
